# Integrating Microfabrication into Biological Investigations: the Benefits of Interdisciplinarity

**DOI:** 10.3390/mi10040252

**Published:** 2019-04-16

**Authors:** Gianluca Grenci, Cristina Bertocchi, Andrea Ravasio

**Affiliations:** 1 Mechanobiology Institute (MBI), National University of Singapore, Singapore 117411, Singapore; 2 Biomedical Engineering Department, National University of Singapore, Singapore 117583, Singapore; 3 Department of Physiology, School of Biological Sciences, Pontificia Universidad Católica de Chile, Santiago 8330025, Chile; cbertocchi@bio.puc.cl; 4 Institute for Biological and Medical Engineering, Schools of Engineering, Medicine and Biological Sciences, Pontificia Universidad Católica de Chile, Santiago 7820436, Chile; andrea.ravasio@uc.cl

**Keywords:** microscopy, microfluidics, microfabrication, biomedical engineering

## Abstract

The advent of micro and nanotechnologies, such as microfabrication, have impacted scientific research and contributed to meaningful real-world applications, to a degree seen during historic technological revolutions. Some key areas benefitting from the invention and advancement of microfabrication platforms are those of biological and biomedical sciences. Modern therapeutic approaches, involving point-of-care, precision or personalized medicine, are transitioning from the experimental phase to becoming the standard of care. At the same time, biological research benefits from the contribution of microfluidics at every level from single cell to tissue engineering and organoids studies. The aim of this commentary is to describe, through proven examples, the interdisciplinary process used to develop novel biological technologies and to emphasize the role of technical knowledge in empowering researchers who are specialized in a niche area to look beyond and innovate.

## 1. Introduction

Our understanding of physiological functions and diseases states, and therefore our ability to develop effective therapeutic strategies, are limited by the overwhelming system-level complexity of biological networks and the spatiotemporal integration of their multiscale and multiparametric components [1]. To deal with such complexity, biologists have developed a large toolbox of complementary approaches. Historically, biologists preferentially adopted reductionist experimental strategies where each experiment aims to test one parameter [2]. Such an approach stringently requires that the on-test biological parameter, being a gene, a protein or a whole biological function, is effectively isolated from its network. Typically, this is implemented by genetic manipulation and by controlling the experimental conditions. Thus, careful design and supervision over the experimental settings are necessary for successfully conduct the experiment and the use of pairwise controls to validate and generalize the conclusions. However, the very same redundancies that provide robustness to life are the major drawback of this strategy as rarely a node in a biological network can be pinpointed by a single manipulation to an unambiguous conclusion [2]. In recent years, more holistic and quantitative approaches to biological research have emerged [3,4,5,6,7]. For instance, synthetic biology and mechanobiology have opened the way for engineering the complexity of biological systems by providing biomimetic models of bioreactions and biological functions [4]. Various microfluidic and on-chip technologies (lab-on-chip, organ-on-chip, body-on-chip) have allowed highly sophisticated, and yet simple, bottom-up strategies to recapitulate intricate biological circuits in a miniaturized and customized fashion [8,9,10,11]. Finally, a family of technologies collectively termed “omics” (i.e., genomics, proteomics, lipidomics, metabolomics and functional omics), combined with multiparametric data analysis, provide an entirely new perspective and technological platform to simultaneously investigate dependent and independent parameters of biological systems [7]. For instance, in genomics, one gene microarray experiment can provide an accurate snapshot of the expression level of thousands of genes, simultaneously. Thanks to the combined technological and scientific efforts mentioned above, clinically important innovations such as personalized medicine and point-of-care diagnostic are becoming part of the everyday experience of doctors and patients [12,13,14]. Despite the existence of diverse approaches, innovation in the biological field is driven by the common need for parallelization, miniaturization, and precise customization of the experiments. To this end, microfabrication and lithographic tools have arguably played an essential role.

In this commentary, we will describe the technology context that drove the current tech revolution in biological research and discuss the factors that enabled such breakthroughs. Thereafter, we will illustrate some of our own key developments that emerged from close interaction and collaboration between scientists of different expertise working under the same roof.

## 2. Development of Microfabrication for Biological Research: A Brief Historical Perspective

For the better part of last 70 years, the main driving force for the development of micro/nano technologies has been the need to scale down the size of micro-components. This has resulted in the development of microchips with increased computational capacity, and usually reduced power consumption. Our generation has witnessed the incredible technological revolution that this race has led to. More interestingly, the development of new technologies and implementation of new approaches for the patterning of small features on a large-scale flat substrate has kept pace with Moore’s law [15]. This was achieved thanks to the use of ultra-violet light (UV) of decreasing wavelength in order to improve features resolution: a 1:1 contact printing scheme was employed first, followed by image projection and step-and-scan exposure schemes that allowed reduction in the size of the image through an optical system (see e.g., [16]). Each time a shift in wavelength or in exposure was introduced, a huge initial investment cost was sustained by manufacturers in order to equip their plants with the latest new technology. Nevertheless, the global market size and the pervasive nature of electronic components in almost every aspect of modern society made the investments worthwhile. The huge push towards developing new patterning strategies that allow for faster and cheaper production of integrated circuits of ever-increasing resolution has not always been successful. Take for instance X-ray lithography (XRL), a good example of a promising technology with less than satisfactory insertion in production lines to date [17]. X-ray photons appear as a natural extension of UV lithography with their extremely short wavelengths (≤ 1 nm), but the technical difficulties in realizing exposure systems for XRL that are suitable for industrial scale application practically stopped any further exploration in that direction [18]. For every example of successful innovation scaled from the lab to the industrial application, many more examples of “failed” technologies can be found. However, those efforts were never entirely wasted, as they contributed to expand the micro/nano-patterning toolbox that we have in our hands.

Today many different, perhaps unintended applications or research fields are benefiting from such developments. One such application is soft lithography as introduced by the seminal work of Whitesides for biological and biomedical purposes [19,20]**.** When soft lithography was first described [21,22]**,** the critical step for rendering these technologies for life-science applications was incidentally already included, as molding involves the use of biocompatible and optically clear material such as poly dimethyl siloxane (PDMS). Thus, soft lithography became popular and since then has paved the way for fabrication of micro-devices for biological and bio-medical applications. As a result of these initial groundbreaking innovations, a large toolbox of enabling technologies has been developed [23]. For instance, microfluidic culture technology associated with tissue engineering has been at the forefront for innovation in cell biology research, as it allows a deeper understanding of physiology and disease [24]. Stem cells and cancer cells are examples of cells whose functions are extremely dependent on their surrounding microenvironment [25,26]. The important factors defining the microenvironment, and thus cell function are: cell-cell and cell-matrix interaction and physicochemical factors such as temperature, pH and mechanical loads. Microfluidics, as compared to conventional culture methods allow precise control of the microenvironment, and thus enable mimicry of the in-vivo milieu. In experiments aimed at differentiation of metastable cells like cancer and stem cells, 3D microfluidic culture models have proven to be a powerful tool to improve the physiological relevance of in vitro models [27,28]. Furthermore, investigation of stem cells and their interactions with their environment has a high potential for translational regenerative medicine and stem cell therapies [29]**.** Another application of 3D microfluidic culture model is the chip-based model for cancer invasiveness. Understanding the intra- and extravasation of cancer cells through biological tissues is important to design effective cancer therapies. Thus, to study the biophysical barriers to the metastatic process, where cancer cells cross cell tissues such as the endothelium or blood-brain barrier is of prime importance [30]. For instance, chip-based models allowed to engineer the formation of an endothelial barrier in a 3D biomimetic environment [31,32,33], which recapitulates the physiological conditions of the process in a controlled manner. Furthermore, hollow structures in a microfluidic platform can be used to mimic the biophysical properties of mammary ducts and blood vessels [34]. Application of microfabrication to study tissue- and organ-level processes (tissues-on-a-chip and organs-on-a-chip) is a growing field of research and could serve as a platform for carrying out tightly controlled, high-throughput drugs toxicity screening studies [8,35,36]. Besides controlling the cellular microenvironment, microfabrication and soft lithography techniques have found excellent uses in imaging biological specimens [37,38,39], cell counting and sorting [40,41,42,43,44,45,46,47], and engineering of micro bioreactors [48,49,50], amongst other applications. Furthermore, soft lithography have allowed to propose and develop revolutionary approaches for biomedical applications; one such example is its impact on diagnostics and global health care enabled by miniaturization, costs reduction and integration of multiple functionalities in portable and reliable platforms [51,52]. Production of nano-particles for drug-delivery is another example [53,54], but the list continues to grow and a comprehensive discussion of all the impactful application of soft-lithography a micro-fabrication methods to the biomedical field is out of scope for this commentary.

## 3. Working in an Interdisciplinary Environment: Bridging Biologists and Engineers

**Figure 1:**
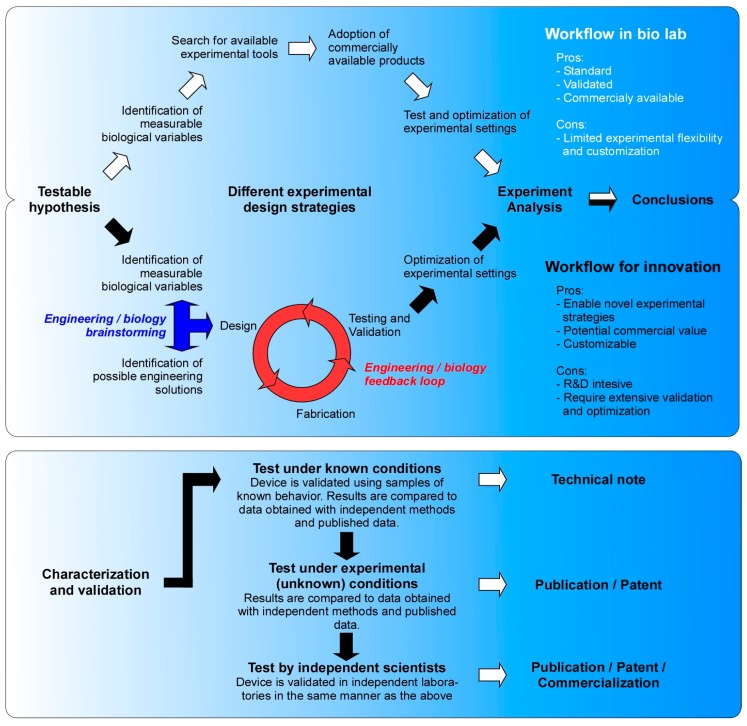
Top panel: Schematic comparison of two different workflows in biological laboratories. Workflow in biomed lab—the path following the empty arrows describes a common experimental approach in biological laboratories relying on commercially available tools. Workflow for innovations—innate inquisitiveness and lack of adequate experimental devices may lead to development of innovative solutions (full arrows). Interdisciplinary team work is often necessary and advisable. Bottom panel: Levels of device characterization and validation (full arrows) and possible outcome as result of successful testing (empty arrows).

As a branch of the life sciences, biological research adopts the scientific method, thereby hypotheses are formulated to address scientific questions and experiments are designed to draw factual conclusions that verify the original hypotheses (Figure 1). In this process, a key bottleneck is the design of adequate experiments that can provide feasible strategies and satisfy rigorous scientific standards. Two courses of action are feasible in this regard: researchers resort to commercially available instrument, devices and packaged kits when available (Figure 1, empty-arrows path). Alternatively, in the absence of commercial solutions, biologists adopt an interdisciplinary approach and devise the necessary enabling tools by collaborating with engineers (Figure 1, full-arrows path). Besides commercial availability, the choice for one or the other solution may also depend on a large variety of parameters such as convenience, time- and cost-effectiveness, feasibility, and the need to customize the experiment, standardization, to name a few. In our experience, the preference for one solution over the other is also influenced by the researcher’s inclination for innovation that leads to development of novel technologies.

Nowadays, researchers have access to an extensive toolbox of instruments, devices and experimental kits that can be purchased from specialized biotech companies. Thus, once they identify the biological parameter that needs to be quantified, scientists can search for commercially available products (Figure 1—top panel, empty arrows path) and adopt them into their research workflow. This path of least resistance offers multiple advantages, such as being time- and cost-effective. Furthermore, mass-produced commercial products are standardized in accordance to international standards for good manufacturing practice and are supplemented with detailed operating protocols. Additionally, commercial solutions go through extensive validation processes carried out by many users over a long period of time, which increases the reliability of the results that they generate and the understanding of possible pitfalls. This allows immediate adoption of the technology by investigators with minimal to no knowledge of the device´s engineering aspects, an ideal situation for biomed specialists who can devote their time to perform experiments and produce quality results. Obviously commercial products also come with their inherent limitations. For example, commercial products typically perform best within a narrow window of specifications. Thus, their applicability is limited to the designated purpose and their customization could be technically challenging, incomplete and ultimately unsatisfactory.

Since biomedicine still remains a frontier science, adoption of lab-ready technologies may not be an option as they may not be available yet. In such cases, an interdisciplinary collaborative approach —combining expertise of scientists in biomedicine and engineering fields—is required to develop the necessary experimental solution (Figure 1—top panel, full arrows path). This typically consists of a two-steps process: firstly, extensive discussion between biologists and engineers must be initiated to identify the biological parameters that need to be quantified and the engineering strategies that could be implemented to develop suitable experimental tools (Figure 1—top panel, blue three-headed arrow). In our own experience, this initial brainstorming step is by far the most challenging part of the process as it requires the translation of biological concepts into feasible engineering strategies and processes. All of this is further complicated by the need to effectively communicate with experts across disciplines, address expectation discrepancies and understand different work style and standards of the various disciplines involved. Therefore, a great deal of interpersonal tolerance, intellectual effort and investment of time for discussions and clarification are key to the success of such collaborative approaches. Next, an iterative process of implementation and optimization is applied during the development and validation of the novel tool (Figure 1—top panel, red three-steps circular workflow). The designing and prototyping steps are critical in finding suitable engineering solutions for the intended biological functionality. In some cases, not often though, already established microfabrication techniques can be directly translated without major variations. Incompatibility of materials, complex sequence of functionalization steps and noncompliance with biological research procedures are all examples of critical designing problems. After successful prototyping, the product is then handed over to the experimentalists to characterize the device performance and validate the results (Figure 1—bottom panel). To choose how to validate the device is a very critical and unintuitive step and it strongly depends on the type of device, its working principle, the intended use and the type of sample. In general, three levels of testing are required for full validation: initially the performance of the device is tested by using sample of well characterized behavior. For instance, polystyrene beads and soluble markers can be used to test the flow in microfluidics [55]. If the performance is not satisfactory, it might be necessary to go back to the drawing board and repeat the previous steps multiple times until optimal performance is achieved. Thereafter, the product’s designated function is probed in real-case biological scenarios of unknown outcome, e.g., by using pairs of treated and untreated samples that allows paired analysis [56]. Those results are then validated using independent experimental conditions and compared to published data for consistency. After this extensive testing phase, the novel experimental device is considered ready for use in its intended scope and it can be disclosed to the public though a publication and the invention protected by a patent. At this point, the device is independently validated by multiple users who serve as beta-tester of the product. If the product performs consistently throughout all the validation process, the innovation may become commercially available and enrich the toolbox available to biologists (Figure 1—top panel, empty arrows path). Obviously, commercialization potential does not necessarily reflect the research value of the innovation as successful commercialization depends on factors like fabrication scalability, size of the potential market, and the intrinsic and perceived monetary value of the product. Once the innovation is made available to the scientific community through publication and/or commercialization, it can be used in any biological lab and hence it enters the workflow described by empty arrows in Figure 1.

## 4. Microfabrication for Biological Investigations: A Few Examples from Our Toolbox

There is a wealth of examples of micro-fabricated devices or methodologies derived from micro-fabrication which have been adapted to bio-medical applications. On one side, we have a long list of researches that take advantage of simple micro-fabrication, where in this context simple is meant as in “established and universally known micro-fabrication procedures”. In Table 1, we propose a short list of such cases. A more extensive discussion comprising of microfluidic examples is out of the scope of this contribution and we kindly refer the readers to reviews on the subject (see for example [57,58,59]).

On the other side, there are those cases where the requirements for the experiment pose a challenge to the micro-fabrication experts and new methodologies or original combinations of established ones need to be developed for the fabrication of the suitable micro-structures. In order to better clarify this claim, in the following sections we will showcase three exemplary cases where newly conceived micro-fabricated devices provided an original platform to address unanswered biological questions. These examples are:

- soSPIM: a light sheet microscopy system that needs only one single objective with high numerical aperture owing to the use of microfabricated disposable chips;
- IR-live: a microfluidic platform for label-free chemical imaging of live cells by infra-red absorption spectroscopy;
- Microfabricated microwells for expansion of circulating tumor cells (CTC).

### 4.1. soSPIM: Advanced Microscopy on A Chip

Selective plane illumination microscopy (SPIM) is a type of fluorescence microscopy which uses a light-sheet to illuminate the sample from the side. The light sheet is normally generated by a low numerical aperture (NA) objective, which is mounted orthogonally to a high NA objective that collects the fluorescent light [84].

In this configuration, planar excitation and the absence of out-of-focus light ensure high contrast, and reduced photo-bleaching and photo-toxicity compared to other fluorescent imaging techniques. However, the requirement for at least two orthogonally aligned objectives makes it difficult to integrate a high NA immersion objective, hindering SPIM use at the single cell level. However such a set-up has been implemented in structured illumination microscopes [85]. Single-molecule detection has also been demonstrated using dedicated and complicated two-objective-based experimental set-ups, e.g., with a movable mirrored cantilever that is placed close to mammalian cells in culture [86] or using lattice light-sheets [87].

**Figure 2:**
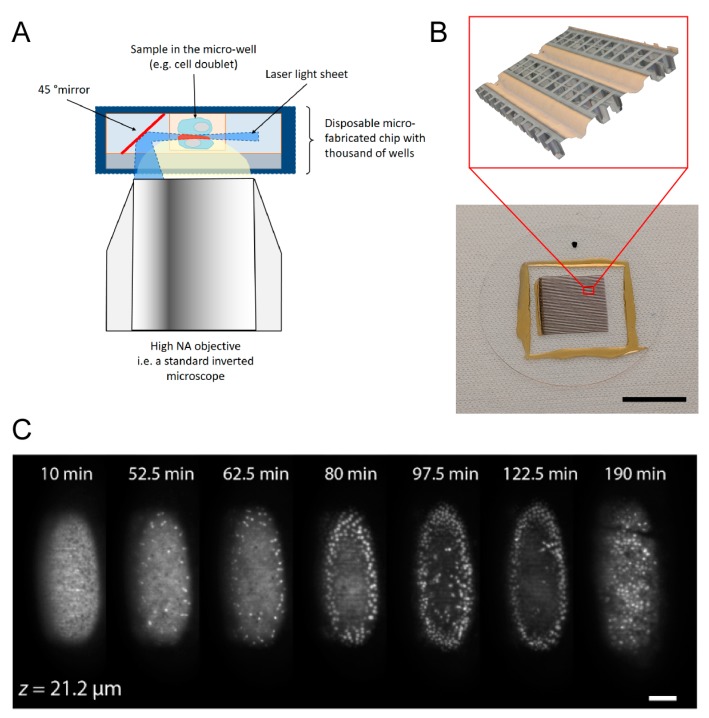
(**A**) Working principle of the soSPIM technique. The excitation laser beam is passed through the same high numerical objective that collect the fluorescent light emitted by the excited sample (shown as a cell doublet in the cartoon). The incoming beam produces a horizontal light sheet by scanning over the surface of the flanking micro-mirror, which is at exactly 45° inclination. By scanning the beam at different height on the mirrors, Z sectioning can be achieved. (**B**) Picture of an actual coverslip (bottom, scale bar is 1 cm) and zoom-in 3D reconstruction image acquired with a Keyence VHX 6000 microscope of the soSPIM device with 40 µm × 40 µm wide micro-wells, 50 µm deep. (**C**) Time-lapse imaging of the early stage of development of a *Drosophila* embryo. Time sequence of soSPIM optical sections 21.2 μm deep within a drosophila embryo expressing the nuclear protein Histone-mCherry imaged with a 20X magnification, 0.5 NA objective and a 4.3 μm thick light-sheet. A 35 μm Z-stack with a 1.35 μm z-step was acquired every 150 seconds for 220 minutes.

Despite its evident benefits, SPIM suffers from the lack of easy solutions for handling samples within the confined space between the two objectives. Observing specimens such as embryos or organoids, with sizes of hundreds of micrometers, is a practical challenge in a standard commercially available SPIM. With this technical problem in mind, a micro-engineering approach was proposed by Galland et al. [88], where a single high NA objective was used for both the generation of the light sheet and the collection of the fluorescent light, giving the instrument its name single-objective SPIM (soSPIM) (Figure 2A). This imaging approach can be implemented on a standard inverted microscope by using disposable cell culture dishes with arrayed micro-mirrors (Figure 2B). The disposable culture dishes are a key component of the soSPIM set-up, which can be exclusively produced using micro-fabrication techniques.

The working principle of the soSPIM (Figure 2A) is simple: micro-mirrors at exactly 45° of inclination are placed next to arrays of micro-wells. The micro-wells (Figure 2B), which can accommodate samples of different size, are the location for cells seeding and observation. The flanking micro-mirrors reflect a laser beam projected from the objective, and a light sheet is generated by horizontally scanning the laser on the mirror surface. Fluorophores in the sample are then excited by this light sheet and images are acquired with the same high NA objective. Sectioning in the Z direction is achieved by producing the light sheet on the mirrors at different planes and by de-focusing to different distances.

The details of the micro-fabrication steps can be found in the original publication [88], but the key aspect is that the disposable micro-mirrored coverslips are produced by a molding procedure starting with a primary mold made of silicon. This primary mold is fabricated through a combination of lithographic and etching processes, taking advantage of the crystal properties of silicon to produce optically smooth mirror surfaces. These surfaces are of the required inclination and can be aligned few micrometers apart from to the multi-well arrays. Moreover, the molding approach enables scaling up the production from handmade laboratory-based to semi-automated industrial, which makes the technique feasible for commercialization. In Figure 2C, we show an example of the potential of soSPIM (reprinted with permission from [88]). The use of larger mirrors adjusted according to the size of the samples, and a horizontal rotation stage for multiview imaging, enabled us to expand the capabilities of our system to perform time-lapse imaging of a *Drosophila* embryo with enhanced long-term stability (thanks to the use of a single objective) and without the requirement of perfect mechanical alignment of the two objectives as in traditional SPIM.

The soSPIM project highlights few of the key elements in the “path for innovations” described in Figure 1. Based on an original realization of an already disclosed microscopy approach, the implementation of this original idea required the design, fabrication and testing of a newly conceived micro-optical device. Once identified the lay-out of the device and its functional requirements (e.g., optical smoothness of the mirroring surfaces, size and arrangement of the micro-wells, materials compatibility), the “Engineering/biological validation” red loop was run across few times before reaching a final protocol capable of producing viable devices in high enough numbers for practical utilization in biological experiments. At this point, internal validation (a still on-going process) was conducted *via* experimental collaboration within the team that developed the soSPIM and colleagues working at the same institution [88,89]. The project is presently being evaluated for possible commercial exploitation with the help of several external groups acting as beta-testers.

### 4.2. IR-live: Infra-red Spectro-microscopy On Live Cells

Infrared (IR) spectroscopy, also known as Fourier transform IR spectroscopy (FTIR), uses the absorption of IR photons as a way to characterize the chemical content of a sample with little to none preparation. Illumination with infrared light promotes energy exchange between the inherent vibrational modes of molecular bonds and incident photons. The exchange results in distinct, fingerprint-like spectral bands that appear in absorption measured as a function of wavelength of incident light, while the energy exchange in the form of heat is negligible. The precise position, line shape, and intensity of infrared absorption bands depend on the molecular structure and conformation as well as intra- and inter- molecular interactions [90].

Despite some major improvements witnessed in recent years, such as the development of bright light sources like Synchrotrons [91] and quantum cascade lasers [92] and the availability of arrays of IR detectors in a configuration similar to that in a CCD camera (IR Focal Plane Arrays (FPA)), biologists have yet to use FTIR for live-cells imaging on a regular basis. And for at least one good reason: the strong water absorption in the mid-IR range. Even layer as shallow as 10 µm thick can completely obscure the features of live-cells due to the characteristic water absorption spectrum in the same IR range where cell’s components are to be found. To tackle this problem, several groups have proposed microfluidic devices [93,94,95] or confined liquid compartments [96] which enable investigations into cellular processes, such as cell death [97], cell cycle [98], stem cell differentiation [99] or protein misfolding [100] at a single cell level and a subcellular spatial resolution [101]. However, the strategies adopted by these groups are often hampered by slow and expensive fabrication processes leading to limited experimental flexibility [102].

**Figure 3:**
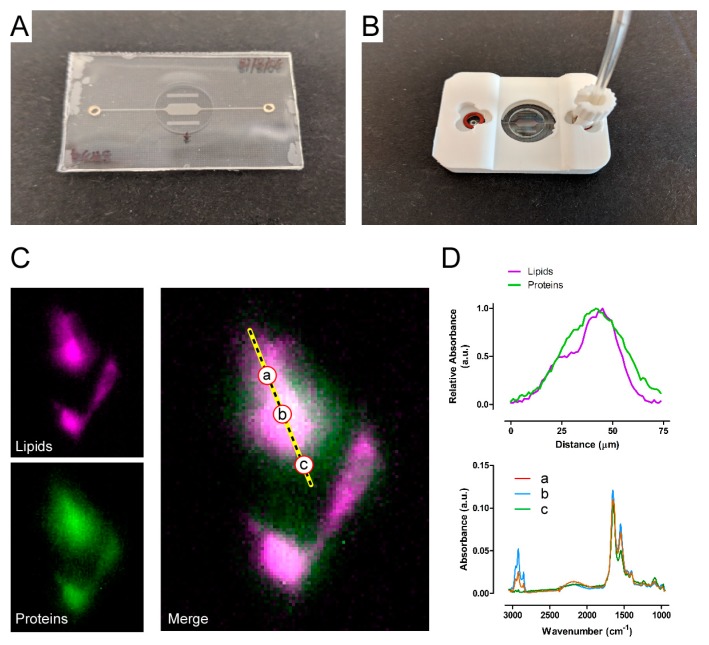
(**A**) A picture of the device for IR spectroscopy. (**B**) The device is shown mounted within the 3D printed plastic jig that allows for easy connection to external fluid management system and that it is compatible with a standard FTIR microscope set-up. (**C**) High-spatial resolution chemical maps of protein (magenta) and lipids (green) as measured in live REF52 cells (re-printed with permission from [55]. (**D**) re-printed with permission from [55]: top panel shows the line profile intensity of proteins (green) and lipids (magenta) as measure along the dashed yellow line in the merged chemical map shown in C. In the bottom panel, 3 punctual absorption spectra are shown for the pixel marked as a, b and c along the same line.

To facilitate the application of FTIR for live-cells imaging there is a need for easy-to-use and standardized microfluidic devices. With this in mind, Birarda et al. [55] proposed and demonstrated a soft-lithographic approach, wherein plastic devices with embedded transparent view ports (CaF_2_ disks with 10 mm diameter and 1 mm thickness) are fabricated. The full fabrication strategy is detailed in [55,103]. It is interesting to highlight that this strategy requires accessing a clean-room micro-fabrication facility only for the generation of a silicon primary mold, which is used as the base for the lay-out of the final device. The actual production of the device can be performed in any laboratory equipped with standard non-lithographic tools (e.g., a plasma system, a hot plate and a UV light). Conveniently, the fabrication of silicon molds can be outsourced to commercial facilities, therefore, in principle, such microfluidic devices can be fabricated with knowledge of only soft lithographic procedures and without an extensive understanding of lithographic processes. Figure 3 shows the resulting device (Figure 3A) with a plastic adaptor for mounting under the microscope (Figure 3B). The standardization provided by the proposed approach, allowed to further explore the design and production of prototypes for the mounting jigs through 3D printing techniques, thanks to the precise positioning of inlet and outlet ports and precise control of the overall dimensions of the devices. This is a step further in the direction of evolving FTIR as a suitable imaging technique for biological applications, as it simplifies the setting up of experiments and does not require advanced skills in handling microfluidic devices.

Using this new kind of devices, live cells IR spectromicroscopy on rat embryo fibroblast cell line REF-52 with high spatial resolution was proven. The chemical maps, line profile and punctual spectra shown in Figure 3C,D are re-printed with permission from [55]. The spatial distributions of two major components of the cells, proteins and lipids, are reconstructed with a pixel size of 1.1 µm × 1.1 µm from spectroscopic data acquired with an FPA at the BSISB program facility (Advanced Light Source beamline 5.4, Berkeley, CA, USA). Details of the data acquisition and analysis procedures are described in the same publication. In short, proteins and lipids content are quantified by calculating the integral below the normalized absorption spectra (as shown in Figure 3D, lower panel) in their respective peak area inside spectral regions: Amide II 1480–1600 cm^−1^ for proteins and 2800–3000 cm^−1^ for lipids.

Looking back to the initial attempts at producing FTIR compatible devices for live cells chemical mapping [104,105], the IR-live project is a good example, in our opinion, of the potential for innovation provided by micro-fabrication and in particular soft-lithography. The utilization of a micro-device in a biological research context is always setting for challenging requirements, such as biocompatibility and suitability for the experimental procedures. Here, a supplement of difficulty is provided by the previously unexplored properties of IR-compatible materials (calcium or barium fluoride, as used in the discussed examples), together with a required attention to a production scheme that would makes it possible for the final user to be independent from an advanced micro-fabrication facility. We argue that moving from the initial standard lithographic to the soft-lithographic approach enables a wider application of FTIR methods to biological research and its requirements, in that it makes easier to design and fabricate microfluidic devices with more advanced functions.

### 4.3. Microfabricated Microwells for Expansion of Circulating Tumor Cells (CTC)

Metastasis is a multi-step process characterized by proliferation of tumor cells, their intravasation into the blood or lymph, followed by their extravasation into the surrounding tissue and subsequent outgrowth in the new microenvironment [106]. Once released from the primary tumor, tumor cells enter the circulatory system; here they can be detected in the bloodstream as circulating tumor cells (CTCs) or in the bone marrow as disseminated tumor cells (DTCs). Since bone marrow sampling is a fairly invasive procedure, it is not generally favored for the clinical management of cancers [107,108]. Thus, in recent years, the focus has been on detecting CTCs in peripheral blood, as there is a clear association of CTCs with metastasis, clinical stage, prognosis of cancers and the response of patients to treatment [109,110]. The detection of CTCs could therefore be a promising minimally invasive diagnostic test for screening patients with metastatic cancers. The main challenge in detecting CTCs is their low levels in blood [111], which makes their recovery from patient samples extremely difficult. Thus, the idea is to design tools that can enrich viable CTCs in the sample for metastatic cancer diagnosis, treatment monitoring, personalized drug screening, and subsequent research studies.

Although several methods for culturing CTCs in vitro have been established in the past [111,112,113,114,115], a few critical issues, such as long culturing time (up to a month), need for pre-enrichment procedures, and low efficiency in CTC isolation (< 20%), still remain to be solved before diagnostic tools based on CTC counts can be routinely used.

Some of these major concerns were addressed in [116], in which the use of culture dishes with tapered micro-wells and an optimized protocol for enrichment were proposed as an improved method for effective CTC isolation. The tapered shape of the micro-wells was identified as one of the key factors contributing to the efficiency of this technique. Indeed, the morphology of the spheroids and CTC clusters created using this approach was found to conform to the micro-well boundaries. Unfortunately, the wells were produced by laser ablation, which resulted in micro-wells with inconsistent morphologies, de facto limiting the accuracy of clusters comparison. Hence, a photolithographic micro-fabrication approach was explored to control the shape of the wells with better precision.

**Figure 4:**
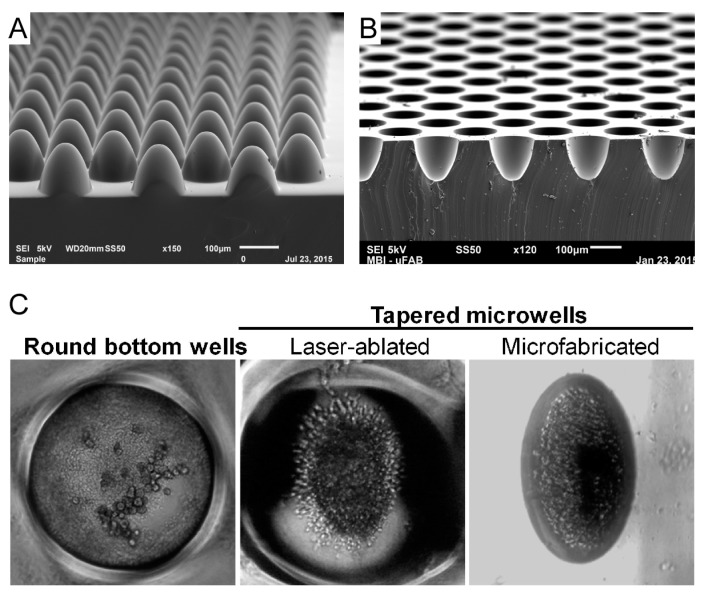
(**A**) SEM images of a PDMS working mold presenting ellipsoidal domes. (**B**) SEM of the PDMS device presenting ellipsoidal wells produced by replica of the mold shown in (**A**). (**C**) Reprinted with permission from [118]. Optical images of resulting CTC cluster in 3 different types of micro-wells. Clinical samples do not form clusters in conventional flat bottom, cylindrical wells (left) but are able to develop clusters consistently in the tapered ones (center and right-side pictures). The ellipsoidal wells produced by micro-fabrication techniques were proven to give the highest efficiency in the production of homogeneous clusters.

This was achieved using a methodology detailed in [117,118,119] that is based on an unusual photolithographic approach, referred to as diffuser back-side lithography [120]. This method allows the fabrication of photo-resist micro-structures (pillars) with rounded profile (Figure 4A) which were considered ideal for this application. By molding a replica of these pillars in a PDMS layer, an array of micro-wells with the designed geometry and distribution, as shown in Figure 4B was generated; a complete device (as presented in the published results) can be produced as an assembly of 3 independently fabricated PDMS functional layers: a gradient generator to produce different concentrations of chemicals of interest, a micro-channels layer that confines the different concentrations and directs them to the layer containing the ellipsoidal micro-wells. As a result, each channel with micro-wells will contain cells clusters subjected to a different chemical mix within the same platform.

It is worth mentioning that the overall fabrication strategy for this 3-layered device is aimed at enabling flexibility in design according to the final application. The micro-wells array can be arranged in as many separated channels and with as many wells as required, while the gradient generator layer can be incorporated with modified designs and functional elements (e.g., pre-mixing, reservoirs, valves layers, and so on) exploiting thus the rich arsenal developed by microfluidic technologies.

Figure 4C is re-printed with permission from [118] and it shows optical microscopy pictures of exemplary CTC clusters as produced in the micro-wells of three different types. In the left-side picture the use of cylindrical micro-wells, i.e., with vertical walls and flat bottom, proved to be inefficient in the production of clusters, while the laser ablated wells (middle picture) are capable of promoting the clustering of the cells but with the limitations already introduced. Micro-fabricated ellipsoidal wells as shown in the right picture give instead consistent clusters with high efficiency.

The key element of this CTC liquid biopsy platform is arguably the design of the micro-wells array. The previous experiments with cylindrical and irregular wells suggested to the researchers the need to explore a system with better defined and homogeneously shaped wells for the growth of the CTC clusters. Under the hypothesis that this would provide for a more efficient control of the biological system, the discussion with the microfabrication team led to a viable strategy for the fabrication of the device, which suitability for clinical purposes is undergoing validation.

## 5. Concluding Remarks

The three examples we have discussed are a small selection of the many instances of fruitful interdisciplinary collaborations that we experienced at the Mechanobiology Institute (MBI). At MBI, researchers with backgrounds as diverse as in engineering and biology work in an open lab environment and share common laboratory facilities. The continuous dialogue that ensues gives rise to a common “mechanobiology” language and encourages sharing of expertise.

Historically, successful scientists used to be ‘jack-of-all-trades’: knowledgeable about science and, at the same time, experienced craftsmen who could build their own experimental tools. For instance, astronomers and microscopists of the past were often skillful craftsmen who showed astonishing degrees of perfection in glass working, producing perfectly shaped mirrors and lenses for their magnifying tubes. Besides legends like Galileo Galilei and Sir Isaac Newton that we instantly think of, several prominent scientists-cum-craftsmen existed throughout the 19th century. Nowadays, this is seldom the case and professional, including scientists and engineers, usually specialize in their own fields, contributing to high throughput, better-quality results. Unfortunately, specialization can sometimes lead to isolation into one’s own area of study. This process has been aggravated by the typical organization of academic institutions into schools and department that are separated by physical structures and independent administrative bodies and by the current status of job markets that are more commonly seeking applicants with specialized skills than those with broader experience.

While the quality and relevance of research conducted by biologists is unquestionable, overspecialization and isolation clearly do not facilitate technical innovation and scientific breakthroughs. As a matter of fact, there are many biological laboratories that routinely use a very limited number of experimental methods and seldom venture into uncharted territories, limiting opportunities for innovation. Therefore, in such units that lack people with diverse skillsets, there is a natural tendency to formulate scientific hypotheses that could be addressed by ‘technology’ preferred by the investigator. This will have a central influence not only in defining the type of experiment that will be conducted, but also the type of questions asked, and hypotheses formulated. Abraham Maslow gets this point across in his law of the hammer: “I suppose it is tempting, if the only tool you have is a hammer, to treat everything as if it were a nail” [121]. Similar to how learning a new language gives the brain new tools for expression, learning and developing new technologies can greatly expand the range of questions and hypotheses put forth by the researcher while investigating a topic of interest.

## 6. Patents

Applications for patent have been filed for the soSPIM project (US 2016/0214107 A1, July 28, 2016) and the CTC liquid biopsy microfluidic assay (WO 2017/188890 A1, November 02 2017).

## Figures and Tables

**Table 1:** Examples of microfabrication technologies applied to biological research.

Application	Method(s)	Required Micro-Fabricated System	References
Protein micro-patterning	Micro-contact printing	PDMS stamps	[60,61,62,63]
	Stenciling	Micro-stencils	[64,65,66,67]
	UV-patterning	UV mask	[68,69,70]
Traction force microscopy	Micro-pillars deflection tracking	Soft micro-pillars	[71,72]
Cells response to topology	Cells culturing on substrates with different topologies	Micro/nano patterned substrates	[73,74,75,76,77]
Collective cells migration	Engineering of environmental cues for collective migration	Micro-stencils Micro-patterning	[78,79,80,81]
Cells response to micro-structured environment	Cells culturing in micro-patterned niches	Substrates with micro-wells	[82,83]
